# Hydrogen gas (H_2_) pretreatment improves lipopolysaccharide-induced acute liver injury in mice by inhibiting NLRP3 inflammasome activation and pyroptosis signaling

**DOI:** 10.3389/fimmu.2026.1759535

**Published:** 2026-03-20

**Authors:** Xinling Chen, Wenting Suo, Qiuling Li, Yun Chen, Yao Deng, Luyao Xu, Jiaying Dai, Ning Zhang, Jiean Xu, Jinwen Xu, Xiaodong Zhang, Wen Su, Chengqin Lu, Shuangling Yang, Hongzhi Yang, Hequan Zhu, Haimei Liu, Wenhai Guo, Yaxing Zhang

**Affiliations:** 1 Department of Physiology, School of Basic Medical Sciences, Guangzhou University of Chinese Medicine, Guangzhou, Guangdong, China; 2 Research Centre of Basic Integrative Medicine, School of Basic Medical Sciences, Guangzhou University of Chinese Medicine, Guangzhou, Guangdong, China; 3 Department of Gynecology, The Second Clinical School of Guangzhou University of Chinese Medicine, The Second Affiliated Hospital of Guangzhou University of Chinese Medicine, Guangdong Provincial Hospital of Chinese Medicine, Guangzhou University of Chinese Medicine, Guangzhou, Guangdong, China; 4 School of Health Sciences, Guangzhou Xinhua University, Guangzhou, Guangdong, China; 5 Department of Traditional Chinese Medicine, The Third Affiliated Hospital, Sun Yat-sen University, Guangzhou, Guangdong, China; 6 Luzhou Key Laboratory of Research for Integrative on Pain and Perioperative Organ Protection, Department of Anesthesiology & Pain, The Affiliated Traditional Chinese Medicine Hospital, Southwest Medical University, Luzhou, Sichuan, China

**Keywords:** acute liver injury, hydrogen gas, innate immunity, lipopolysaccharide, NLRP3 inflammasome, pyroptosis, sepsis

## Abstract

**Background:**

The liver is extremely vulnerable to endotoxin-induced damage during sepsis. Hydrogen gas (H_2_) is a colorless and odorless gas molecule with anti-oxidative and anti-inflammatory actions. However, the effects of H_2_ intraperitoneal injection on sepsis-induced acute liver injury and the possible mechanisms remain unclear.

**Methods:**

Biochemical analysis, H&E staining, immunoblotting, immunofluorescence, and TUNEL staining were used to investigate the effects and mechanisms of H_2_ intraperitoneal injection on lipopolysaccharide (LPS)-induced acute liver injury in mice. AML12 cells and pharmacological rescue experiment were used to confirmed the target of H_2_.

**Results:**

H_2_ pretreatment by intraperitoneal injection improved LPS-induced acute liver injury in mice as indicated by reducing inflammatory cells infiltration in the liver, down-regulating serum ALT and AST levels, decreasing hepatic 3-nitrotyrosine, MDA, and MPO levels, and up-regulating hepatic GSH levels. Mechanistically, H_2_ suppressed TLR4 to IKK-NF-κB and to MAPK (ERK, p38 and JNK) signaling, and thus reducing pro-inflammatory cytokines, including TNF-α, IL-1β, and IL-18 levels in the liver of LPS-challenged mice. Moreover, the hepatic pyroptosis signaling including NLRP3 inflammasome (NLRP3, ASC, and Caspase-1) to GSDMD, Caspase-8/11 to GSDMD, Caspase-3 to GSDME, and TUNEL staining in LPS-challenged mice were all reversed by H_2_ treatment. The pharmacological rescue experiments by agonist (nigericin) and antagonist (MCC950) of NLRP3 further confirm the action of H_2_ on NLRP3 *in vitro*.

**Conclusions:**

H_2_ pretreatment by intraperitoneal injection alleviated LPS-induced acute liver injury in mice by modulating redox homeostasis, TLR4-mediated innate immune signaling, NLRP3 inflammasome activation and pyroptosis signaling.

## 1 Introduction

Sepsis is a life-threatening muti-organ dysfunction caused by a dysregulated host response to infection (1). In sepsis, immune response that is initiated by the invading pathogens fails to return to homeostasis, and thus, culminate in a pathological syndrome that is characterized by sustained excessive inflammation and immune suppression (2). The liver is essential to modulate many physiological and pathophysiological processes, including detoxification, metabolism, and immunity, which makes this organ extremely vulnerable to endotoxin-induced damage during sepsis (3–5). The inflammatory response in sepsis is dependent upon activation of pathogen recognition receptors (i.e. Toll-like receptors [TLRs]) in the liver, which are crucial to the pathogenesis of sepsis and sepsis-associated liver injury (6). One of these endotoxins is lipopolysaccharide (LPS), which is a component of Gram-negative bacteria, it induces acute inflammation by binding its receptor TLR4 in its targeting organs to produce proinflammatory cytokines (7). LPS-induced acute liver injury mouse model is widely used for pathogenesis studies, new targets discovery, and drug development for sepsis and its related organ injuries. Although sepsis-related mortality across the majority of analyzed nations between 1985 and 2019 was decreased (8), sepsis and septic shock are still major healthcare problems, impacting millions of people around the world each year and killing between one in three and one in six of those it affects (9). Therefore, it is urgent to search effective therapies and the promising candidate drugs for patients with sepsis.

Hydrogen gas (H_2_) is a strong anti-oxidative agent with anti-inflammatory action. However, endogenous H_2_ in mammalian is mainly produced by hydrogenases-containing microorganism as that mammalian cells without functional hydrogenase genes. Supplement with exogenous H_2_*via* inhalation of H_2_-O_2_ mixture, drinking H_2_-rich water, and injection of H_2_-rich saline showed hepatic protection for improving metabolic dysfunction-associated steatotic liver disease (MASLD) and ischemia-reperfusion (I/R)-induced liver injury (10). However, whether intraperitoneal injection of H_2_ has a therapeutic effect on these diseases is unclear. Previously, we had found that intraperitoneal injection of H_2_ can improve lipopolysaccharide (LPS)-induced cardiac dysfunction (11), alleviate acute ethanol binge-induced acute liver injury (12) and methionine- and choline-deficient diet-induced MASLD in mice (13). Here, LPS-induced acute liver injury mouse model was used to mimic sepsis-induced liver injury, and the effects of H_2_ pretreatment by intraperitoneal injection on acute liver injury was investigated in this model. If it can, we will further examine the possible innate immune molecular mechanisms involved in this process.

## 2 Materials and methods

### 2.1 Animal model of lipopolysaccharide -induced acute liver injury and treatment protocol

Male C57BL/6 mice (21-23g) used in this study were brought from Animal Experiment Center of Guangzhou University of Chinese Medicine. All animals were housed in a temperature-controlled animal facility with a 12-h light–dark cycle and allowed to obtain rodent chow and water ad libitum. All animals were provided with humane care and all animal experiment procedures were approved by the Institutional Animal Care and Use Committee of Guangzhou University of Chinese Medicine. The animal studies are reported in accordance with ARRIVE (Animal Research: Reporting of *In Vivo* Experiments) guidelines 2.0 (14, 15).

**Figure 1:**
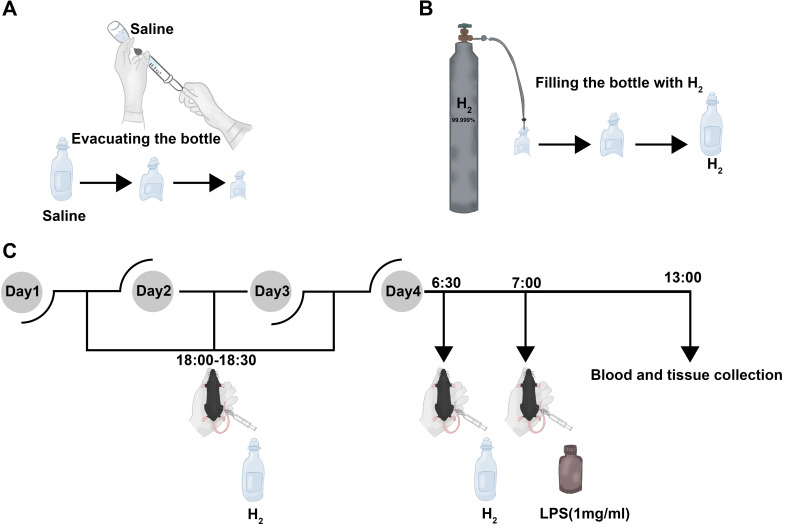
The method of H_2_ intraperitoneal injection and experimental process. **(A)** The sterile saline plastic infusion bottle (100 mL) was evacuated by 50 mL sterile injection syringe. **(B)** The pressure reducing valve in the aluminum high-pressure gas tank was opened with a small release pressure, which purges residual air from the plastic tubing and the syringe needle connected to the pressure-reducing valve. Then, the pressure reducing valve was further loosen and filled the infusion bottle with H_2_ until there is no dead volume. **(C)** The animals in H_2_ treatment group are intraperitoneally injected with H_2_ (0.5 mL/100 g as the low dose and 1.0 mL/100 g as the high dose) at 18:00 - 18:30 daily for the first three days. The animals in LPS group and treatment group are intraperitoneally injected with LPS (5 mg/1000 g) after 30 min of the last H_2_ injection on day 4. Six hours of LPS administration, animals were deeply anesthetized before blood collection from the orbital sinus into microcentrifuge tubes at room temperature for serum collection and euthanized *via* cervical dislocation. In detail, there was a two-way valve connected between syringe and injection needle. The two-way valve was opened, inserted the syringe needle into the H_2_ bottle, and the required volume of H_2_ was drawn into the syringe, then, immediately closed the two-way valve and removed the needle from the infusion bottle. Last, the needle was quickly inserted into the animal’s abdominal cavity, and reopened the two-way valve to complete H_2_ intraperitoneal injection.

Briefly, LPS-induced acute liver injury mouse model was established by a single intraperitoneal injection of LPS (5 mg/1000 g, L2880, Sigma-Aldrich; MerekKGaA, Darmstadt, Germany) dissolved with normal saline (1 mg/mL). The animals in LPS+H_2_ (L) group and LPS+H_2_ (H) group were intraperitoneally injected with H_2_ (99.999%, Guangzhou Gas Group Co., Ltd., Guangzhou, China) packed in an aseptic infusion bag (11) at doses of 0.5 mL/100 g and 1.0 mL/100 g, respectively, once daily for 4 consecutive days. On day 4, after adding H_2_ for half an hour, mice in LPS+H_2_ (L) group, LPS+H_2_ (H) group, and mice in LPS group were intraperitoneally injected with LPS. The animals in Control group were given same volume of normal saline. After 6 hours of LPS administration, animals were deeply anesthetized before blood collection from the orbital sinus into microcentrifuge tubes at room temperature for serum collection and euthanized *via* cervical dislocation (16) (**Figure 1**). The liver samples were harvested, either frozen in -80 °C or put in 4% Paraformaldehyde Fix Solution (G1101, Servicebio, Wuhan, Hubei, China) before further analysis.

### 2.2 Biochemical analysis

The collected blood samples were centrifuged (3500 rpm) for 15 minutes. The biochemical analysis of serum alanine aminotransferase (ALT) and aspartate aminotransferase (AST) were performed as we previously described (17). The hepatic malondialdehyde (MDA), myeloperoxidase (MPO), and reduced glutathione (GSH) were measured according to the instructions of commercial reagent kits from Nanjing Jiancheng Bioengineering Institute, Nanjing, Jiangsu, China (A003-1-2, A044-1-1, and A006-2-1).

### 2.3 H&E staining

For histopathology analysis, paraffin sectioning and hematoxylin and eosin (H&E) were performed according the standard techniques: (1) The liver samples were fixed in 4% Paraformaldehyde Fix Solution. (2) The samples were placed in an embedded box and dehydrated using a dehydrator (Excelsior AS, Leica, Germany). (3) Paraffin embedding is rapidly frozen and stored overnight at -20 °C. (4) The liver tissues were cut into paraffin sections with a thickness of approximately 4 μm using a paraffin microtome (RM2245, Leica, Germany). (5) H&E staining was performed and sealed with neutral resin for observation using an image acquisition system.

### 2.4 Immunoblotting

Total protein was extracted from the liver tissues using the lysis buffer (18), and a commercial Pierce BCA Protein Assay Kit (Thermo Fisher Scientific, 23225) was used to quantified proteins. The proteins were separated using SDS-polyacrylamide gels (PAG) electrophoresis and transferred onto polyvinylidene fluoride (PVDF) membranes (Millipore, IPVH00010), which were incubated with primary and secondary antibodies (**Table 1**) by standard techniques as we had previously described (18). The proteins were visualized with an automatic chemiluminescence instrument (Tanno 5200, Shanghai Tanon Life Science Co., Ltd.) by using Enhanced Chemiluminescent (NCM Biotech, P10300). The Image J software was used to quantify the gray value of the proteins. The molecular weight on the right side of the band is labeled according to the molecular weight of the target band described in the antibody instructions (**Table 1**).

**Table 1:** The information of antibodies.

The antibodies	The product number	Dilution	The manufacturers
Tumor necrosis factor α (TNF-α) antibody	#AF7014	1:1000	Affinity Biosciences (Changzhou, Jiangsu, China)
Interleukin 1β (IL-1β) antibody	#AF5103	1:1000	
TMS1/ASC antibody	#DF6304	1:2000	
Goat Anti-Rabbit IgG (H+L) HRP	#S0001	1:4000	
Goat Anti-Mouse IgG (H+L) HRP	#S0002	1:3000	
TLR4 antibody	#sc-293072	1:2000	Santa Cruz Biotechnology (Santa Cruz, CA, USA)
Caspase-3 antibody	#sc-56053	1:2000	
Caspase-11 antibody	#sc-374615	1:2000	
Phosphorylated NF-κB p65 antibody	#3033S	1:2000	Cell Signaling Technology (Danvers, MA, USA)
NF-κB p65 antibody	#8242S	1:2000	
Erk1/2 antibody	#4695S	1:1000	
Phosphorylated p38 antibody	#4511S	1:2000	
p38 antibody	#8690S	1:2000	
Phosphorylated IKKα/β antibody	#2697S	1:2000	
IKKβ antibody	#8943S	1:2000	
NLRP3 antibody	#15101S	1:2000	
JNK antibody	#9252S	1:1000	
Phosphorylated JNK antibody	#9255S	1:2000	
Caspase-8 antibody	#9746S	1:2000	
Phosphorylated ERK1/2 antibody	#bs-3016R	1:1000	Bioss Antibodies (Beijing, China)
3-nitrotyrosine (3-NT) antibody	#ab110282	1:1000	Abcam (University of Cambridge, UK)
Caspase-1 antibody	#ab1872	1:2000	
GSDMD antibody	#ab219800	1:2000	
GSDME antibody	#ab215191	1:2000	
IL-18 antibody	#D046-3	1:1000	Medical & Biological Laboratories Co., Ltd (Tokyo, Japan)
GAPDH antibody	#MB001	1:10000	Bioworld Technology (Qixia District, Nanjing, China)

### 2.5 Immunofluorescence staining

The liver tissues were fixed in 4% Paraformaldehyde Fix Solution for 24 hours, and then, gradually dehydrated in 15% sucrose solutions and 30% sucrose solutions for 24 hours, respectively. The samples were embedded in a frozen section embedding matrix (BL557A, Biosharp, Hefei, China), and cut into slices on a cryostat at -20 °C. The prepared slices were washed with 1 × PBS for 30 min, 1 × TBS for 10 min, and 1 × TBST for 10 min, respectively. Then, the slices were incubated with sheep serum for 2 hours at room temperature, subsequently, incubated with 3-NT antibody overnight. The slices were further washed with 1 × TBS for 10 minutes and 1 × TBST for 20 min, and then, incubated with secondary antibody for two hours at room temperature in the dark. After washing the slices for three times with 1 × TBS (10 minutes each time), the anti-fluorescence quenching sealing agent (Solarbio, S2110, Beijing, China) was added, and then, observed 3-NT by a Zeiss inverted fluorescence Microscope. The steps for GSDMD immunofluorescence are similar to those for 3-NT, except that the primary antibody is changed from 3-NT to GSDMD, and the secondary antibody is changed from Goat Anti-Mouse IgG (H+L) Fluor 594-conjugated to Goat Anti-Rabbit IgG (H+L) Fluor 488-conjugated.

### 2.6 TUNEL staining for the liver samples

TUNEL staining was performed using a commercial TUNEL assay kit (E-CK-A320, Elabscience, Wuhan, China) according to the following protocol: Paraffin-embedded sections were deparaffinized in xylene twice (10 min each), followed by soaked in absolute ethanol twice (5 min each). The sections were further soaked in 90%, 80%, and 70% ethanol aqueous solutions (3 min each). Subsequently, the sections were washed in PBS for three 5-minute, and residual liquid around the samples was blotted with filter paper. Each sample was treated with 100 μL of 1× Proteinase K working solution (Proteinase K: PBS = 1:99) and incubated at 37 °C for 20 min. Following three additional PBS washes (5 min each), 100 μL of TdT Equilibration Buffer was added to each sample and incubated at 37 °C for 20 min, and the buffer was removed by blotting with absorbent paper. Then, 50 μL of labeling working solution (TdT Equilibration Buffer: Labeling Solution: TdT Enzyme = 35: 10: 5) was applied to each sample and incubated in the dark at room temperature for 60 min. The samples were washed in PBS for three 5-minute. Finally, the sections were mounted with an anti-fade mounting medium containing DAPI (Cat# S2110, Solarbio, Beijing, China), and fluorescence intensity was observed under a fluorescence microscope.

### 2.7 Cell culture and pharmacological rescue experiment

AML12 cells, which was established from hepatocytes from a mouse (CD1 strain, line MT42) transgenic for human TGF alpha, were obtained from Procell Life Science & Technology Co., Ltd (CL-0602, Wuhan, China). Hepatocytes were cultured in AML12-specific medium (CM-0602, Procell Life Science & Technology Co., Ltd, Wuhan, China) including DMEM/F12 + 10% FBS + 0.5% ITS-G (100×) + 40 ng/mL Dexamethasone + 1% P/S under conditions of 37 °C and 5% CO_2_. The hepatocyte suspension (100 μL/well) was inoculated into 96-well plate until the cell density reached approximately 80%. Each plate set up three blank wells with culture medium but without cells. Hepatocytes were starved in AML12-specific medium containing 1% FBS for 24 hours. H_2_-rich medium was pre-treated for 30 minutes. Then, 1 μg/mL LPS was added for 4 hours, subsequently, 5 μM NLRP3 agonist nigericin was added for 2 hours (19); or hepatocytes were treated with 1 μg/mL LPS for 5 hours and subsequent 5 μM NLRP3 inhibitor MCC950 for one hour (20). After finishing the treatment, 10 μL CCK-8 solution was added under light-avoiding conditions, the 96-well plates were wrapped by aluminum foil and incubated for one hour. Finally, the absorbance (A) was measured at 450 nm using a microplate reader, and cell viability (%) = [(A (Dosing)- A (Blank)/(A(Control)- A (Blank)]*100%.

### 2.8 Statistical analysis

Statistical analyses were performed by one-way analysis of variance (ANOVA) followed by Bonferroni’s *post hoc* analysis for data with normal distribution (by Shapiro-Wilk test) and homogeneity of variance (by Brown-Forsythe test). If data with normal distribution and heteroscedasticity, statistical analyses were performed by Brown-Forsythe and Welch ANOVA tests followed by Dunnett T3 *post hoc* analysis. All data were expressed as mean ± SD, a value of P < 0.05 was considered as significantly different. All histograms were performed using GraphPad Prism 9.3.0 (GraphPad Software Inc., San Diego, CA, USA).

## 3 Results

### 3.1 H_2_ pretreatment by intraperitoneal injection alleviates LPS-induced acute liver injury in mice

**Figure 2:**
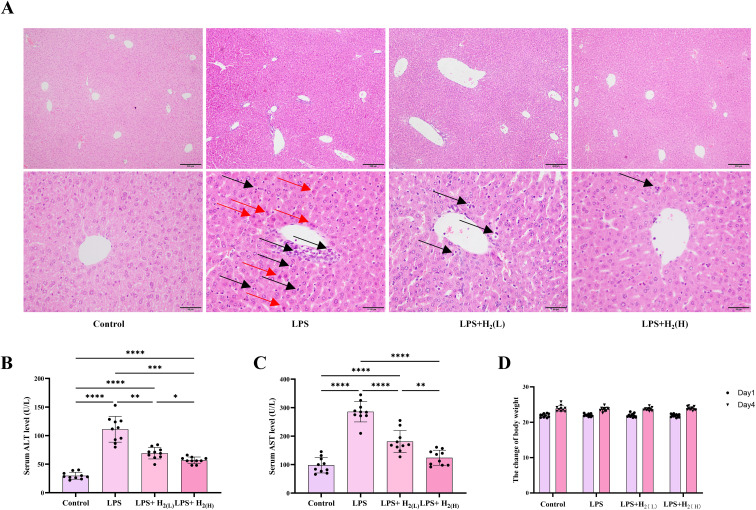
H_2_ pretreatment alleviates LPS-induced acute liver injury in mice.**(A)**. The liver H&E staining, the scale bars of the upper images and the lower image are 200 µm and 50 µm, respectively. The red arrow represents denatured and necrotic liver cells, and the black arrow represents the infiltrated inflammatory cells. **(B)**. The serum ALT levels. **(C)**. The serum AST levels. **(D)**. The body weight before and after the experiment. n = 10 biologically independent samples in each group, ^*^p <0.05, ^**^p < 0.01, ^***^p < 0.001, ^****^p < 0.0001. The horizontal line indicates that the group on the left is compared with the group on the right.

The liver is one of the major affected organs in sepsis. Here, we first investigated the effect of H_2_ pretreatment by intraperitoneal injection on LPS-induced acute liver injury in mice. Compared with Control group, LPS induced eosinophilic degeneration and necrosis of hepatocytes, increased infiltration of inflammatory cells in pericentral vein and intrahepatic sinuses (**Figure 2A**). H_2_ pretreatment improved these pathological changes, and the high-dose H_2_ was more effective than the low-dose (**Figure 2A**). Moreover, H_2_ pretreatment decreased serum ALT and AST levels when compared with LPS group (**Figure 2B, C**). However, the ALT levels in two treatment groups and the AST levels in LPS + H_2_ (L) group were still higher than those in Control group. The ALT and AST levels in LPS + H_2_ (H) group were lower than these in LPS + H_2_ (L) group, and there was no difference in AST levels between LPS + H_2_ (H) group and Control group. Moreover, there were no statistically significant differences in body weight among the groups, either before or after the experiment (**Figure 2D**). Therefore, H_2_ pretreatment by intraperitoneal injection has a preventive effect on LPS-induced acute liver injury in mice.

### 3.2 H_2_ pretreatment suppresses oxidative stress in the liver of LPS-challenged mice

**Figure 3:**
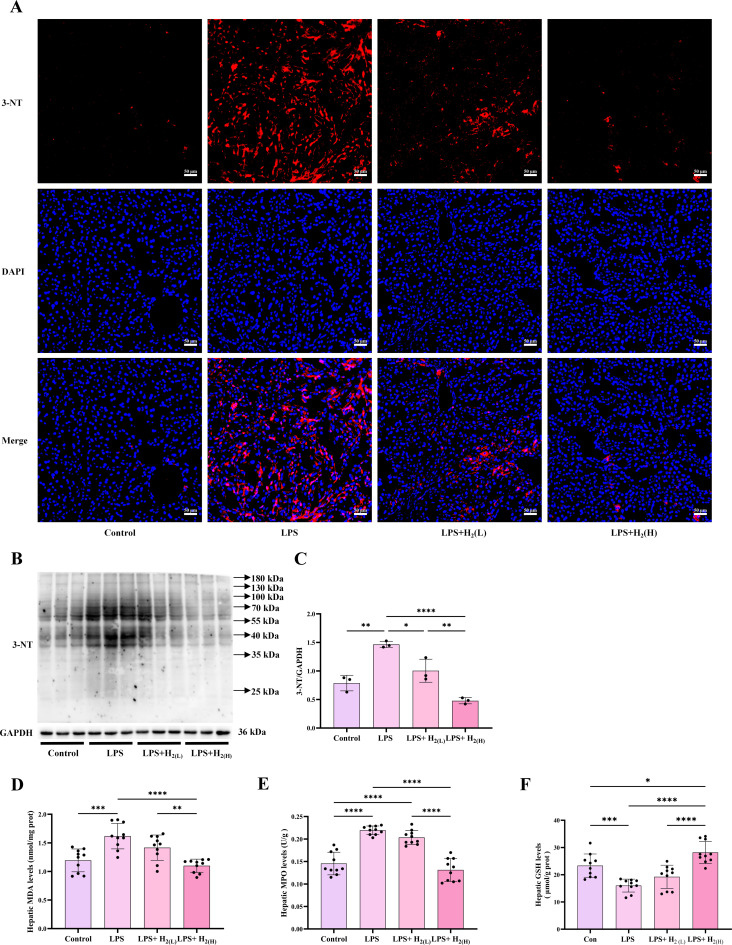
H_2_ pretreatment improves LPS-induced hepatic oxidative stress in mice. **(A)** The immunofluorescence of 3-NT in the liver (scale bars: 50 µm). **(B)** The immunoblotting images of 3-NT and GAPDH in the liver and **(C)** the quantification of 3-NT to GAPDH ratio, n = 3 biologically independent samples. **(D)** The MDA levels, **(E)** MPO levels, and **(F)** GSH levels in the liver, n = 10 biologically independent samples. ^*^p <0.05, ^**^p < 0.01, ^***^p < 0.001, ^****^p < 0.0001. The horizontal line indicates that the group on the left is compared with the group on the right.

LPS can increase the production of reactive oxygen species (ROS) to enhance oxidative stress in the liver (21). 3-NT is one of the biomarkers to reflect oxidative stress (22). Both immunofluorescence staining and immunoblotting showed that hepatic 3-NT levels were increased by LPS, these were decreased by H_2_ pretreatment, and high-dose H_2_ has a better effect (**Figures 3A–C**). ROS can directly damage lipids, and MDA is a convenient biomarker to reflect lipid peroxidation (23). Our results showed that hepatic MDA levels were increased in LPS group when compared with Control group, while these were reversed by high-dose H_2_ pretreatment (**Figure 3D**). MPO, the member of heme peroxidase family in immune cells, can generate powerful oxidizing species including hypochlorous acid (HOCl) (24), which also reflects the degree of inflammation and oxidative stress in the tissue or organ. Therefore, we further detected MPO levels in the liver. Compared with Control group, hepatic MPO level were increased by LPS, while these were decreased in LPS+H_2 (H)_ group (**Figure 3E**). In contrast to these substances causing oxidative damage, GSH, which can reduce H_2_O_2_ to H_2_O, is a molecule that reflects antioxidant capacity (25). The hepatic GSH levels were reduced in LPS group compared with Control group, while this down-regulation of GSH was increased by high-dose H_2_ (**Figure 3F**). Therefore, H_2_ pretreatment by intraperitoneal injection can maintain redox homeostasis of the liver in LPS-challenged mice.

### 3.3 H_2_ pretreatment inhibits over-activation of TLR4-mediated innate immune signaling in the liver of LPS-challenged mice

**Figure 4:**
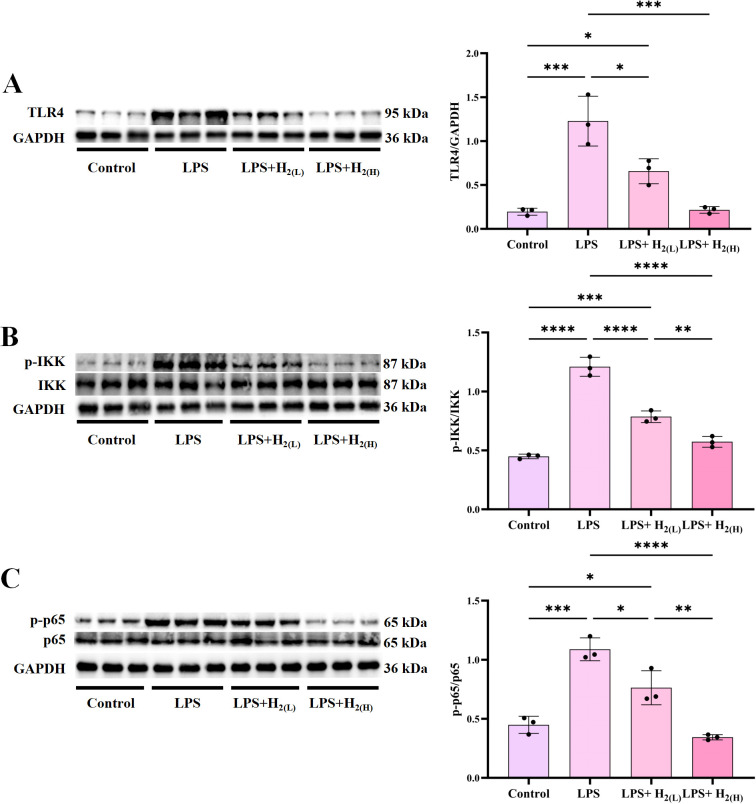
H_2_ pretreatment inhibits TLR4-IKK-NFκB innate immune signaling in the liver of LPS-challenged mice. **(A)** The immunoblotting images of hepatic TLR4 and GAPDH, and the quantification of TLR4 to GAPDH ratio. **(B)** The immunoblotting images of p-IKK, IKK and GAPDH in the liver, and the quantification of p-IKK to IKK ratio. **(C)** The immunoblotting images of hepatic p-p65, p65 and GAPDH, and the quantification of p-p65 to p65 ratio. n = 3 biologically independent samples, ^*^p <0.05, ^**^p < 0.01, ^***^p < 0.001, ^****^p < 0.0001. The horizontal line indicates that the group on the left is compared with the group on the right.

**Figure 5:**
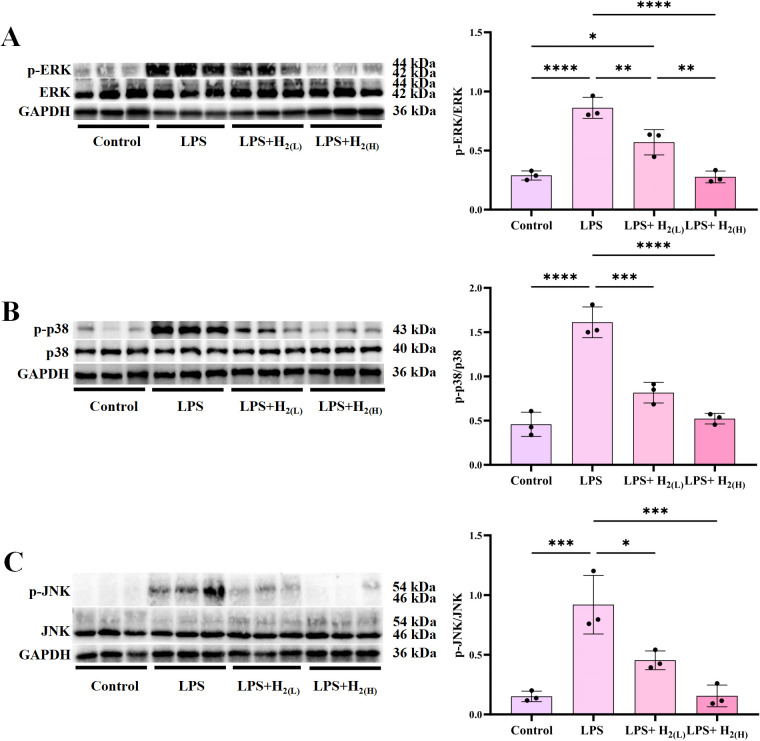
H_2_ pretreatment inhibits hepatic MAPK signaling in LPS-challenged mice. **(A)** The immunoblotting images of hepatic p-ERK1/2, ERK1/2 and GAPDH, and the quantification of p-ERK1/2 to ERK1/2 ratio. **(B)** The immunoblotting images of hepatic p-p38, p38 and GAPDH, and the quantification of p-p38 to p38 ratio. **(C)** The immunoblotting images of hepatic p-JNK, JNK and GAPDH, and the quantification of p-JNK to JNK ratio. n = 3 biologically independent samples, ^*^p <0.05, ^**^p < 0.01, ^***^p < 0.001, ^****^p < 0.0001. The horizontal line indicates that the group on the left is compared with the group on the right.

LPS (also known as endotoxin), a key component of the outer membrane in Gram-negative bacteria, is recognized *via* TLR-4 on cell membranes, and over-activation of TLR4 signaling plays a crucial role in the pathogenesis of sepsis (26). To investigate the effect of intraperitoneal injection of H_2_ on TLR4 signaling in the liver of LPS-challenged mice, immunoblotting was used to examine the expression or activation of signal proteins in TLR4 signaling, including TLR4, its downstream signaling proteins (IKK, NF-κB p65, ERK1/2, p38 MAPK and JNK). LPS up-regulated TLR4 expression (**Figure 4A**), and enhanced the phosphorylation of IKK (**Figure 4B**) and NF-κB (**Figure 4C**), these up-regulation were inhibited by H_2_, and the high dose is better than the lower one. The phosphorylation of ERK1/2, p38 MAPK and JNK were all increased by LPS, in contrast, both low-dose and high-dose H_2_ can inhibit MAPK phosphorylation, and the high-dose is better than that of the low-dose (**Figure 5**). Therefore, H_2_ pretreatment by intraperitoneal injection inhibits over-activation of TLR4-mediated signaling in the liver of LPS-challenged mice.

### 3.4 H_2_ pretreatment inhibits pro-inflammatory cytokines expression and cleavage in the liver of LPS-challenged mice

**Figure 6:**
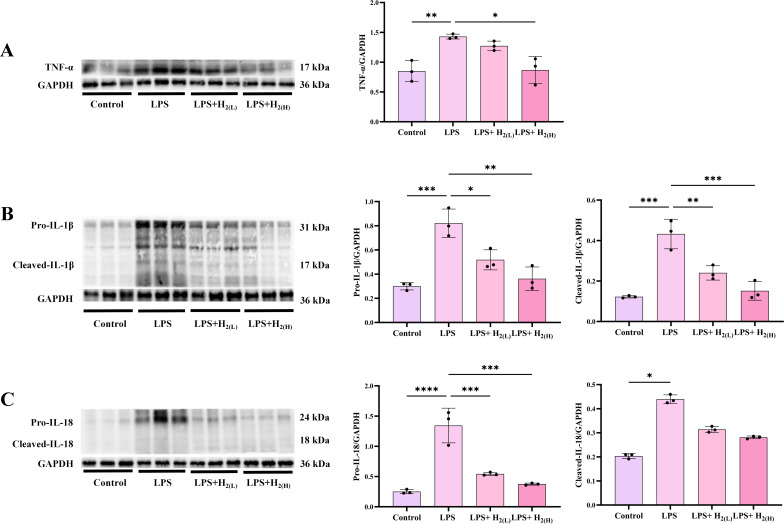
H_2_ pretreatment suppresses pro-inflammatory cytokines expression and cleavage in the liver of LPS-challenged mice. **(A)** The immunoblotting images of hepatic TNF-α and GAPDH, and the quantification of TNF-α to GAPDH ratio. **(B)** The immunoblotting images of hepatic pro-IL-1β, cleaved-IL-1β and GAPDH, and the quantifications of pro-IL-1β to GAPDH and cleaved-IL-1β to GAPDH ratios. **(C)** The immunoblotting images of hepatic pro-IL-18, cleaved-IL-18 and GAPDH, and the quantification of pro-IL-18 to GAPDH and cleaved-IL-18 to GAPDH ratios. n = 3 biologically independent samples, ^*^p <0.05, ^**^p < 0.01, ^***^p < 0.001, ^****^p < 0.0001. The horizontal line indicates that the group on the left is compared with the group on the right.

Over-activation of TLR4-mediated innate immune signaling can cause excessive production of TNF-α, IL-1β, and IL-18, thus amplifying inflammation and oxidative damage (27, 28). Here, the levels of hepatic TNF-α, pro-IL-1β, cleaved-IL-1β, pro- IL-18, and cleaved-IL-18 were all increased by LPS, in contrast, the expression and cleavage of these pro-inflammatory cytokines (except cleaved-IL-18 with no significance) were all reduced by H_2_ pretreatment (**Figure 6**). Therefore, H_2_ pretreatment by intraperitoneal injection suppresses pro-inflammatory cytokines expression and cleavage in the liver of LPS-challenged mice.

### 3.5 H_2_ pretreatment suppresses NLRP3 inflammasome activation and pyroptosis in the liver of LPS-challenged mice

**Figure 7:**
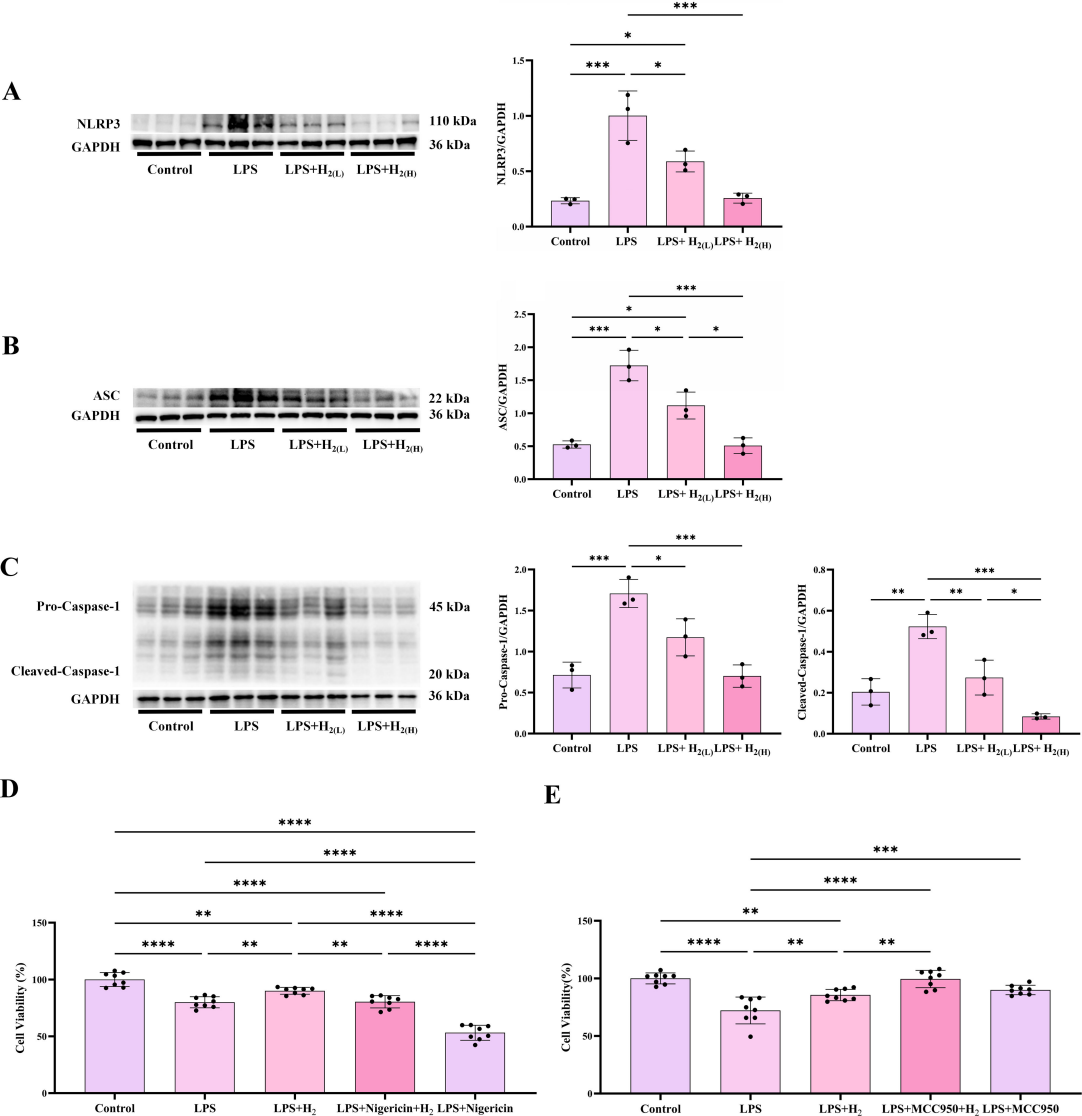
H_2_ pretreatment inhibits NLRP3 inflammasome activation both in LPS-challenged mice and in cultured hepatocytes. **(A)** The immunoblotting images of hepatic NLRP3 and GAPDH, and the quantifications of NLRP3 to GAPDH ratio. **(B)** The immunoblotting images of hepatic ASC and GAPDH, and the quantifications of ASC to GAPDH ratio. **(C)** The immunoblotting images of hepatic pro-Caspase-1, cleaved-Caspase-1 (p20 subunit) and GAPDH, and the quantifications of pro-Caspase-1 and cleaved-Caspase-1 to GAPDH ratios. **(D)** The pharmacological rescue experiments by agonist (nigericin) of NLRP3 *in vitro*. **(E)** The pharmacological rescue experiments by antagonist (MCC950) of NLRP3 *in vitro*. n = 3 biologically independent samples *in vivo* and n = 8 biologically independent samples *in vitro*, ^*^p <0.05, ^**^p < 0.01, ^***^p < 0.001, ^****^p < 0.0001. The horizontal line indicates that the group on the left is compared with the group on the right.

**Figure 8:**
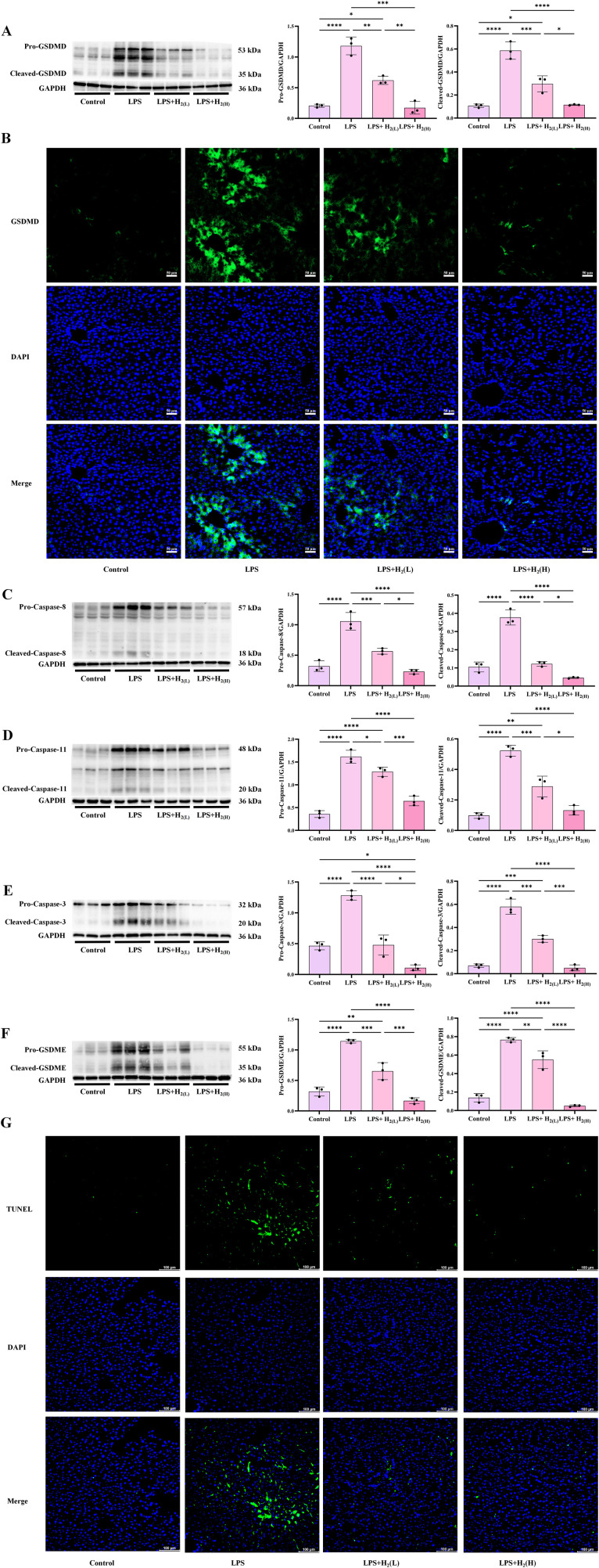
H_2_ pretreatment inhibites pyroptosis signaling in the liver of LPS-challenged mice. **(A)** The immunoblotting images of hepatic pro-GSDMD, cleaved-GSDMD and GAPDH, and the quantifications of pro-GSDMD to GAPDH and cleaved-GSDMD to GAPDH ratios. **(B)** The immunofluorescence staining of GSDMD (scale bars: 50 µm). **(C)** The immunoblotting images of hepatic pro-Caspase-8, cleaved-Caspase-8 (p18) and GAPDH, and the quantifications of pro-Caspase-8 to GAPDH and cleaved-Caspase-8 to GAPDH ratios. **(D)** The immunoblotting images of hepatic pro-Caspase-11, cleaved-Caspase-11 (Caspase-11 p20 subunit) and GAPDH, and the quantifications of pro-Caspase-11 to GAPDH and cleaved-Caspase-11 to GAPDH ratios. **(E)** The immunoblotting images of hepatic pro-Caspase-3, cleaved-Caspase-3 (Caspase-3 p20) and GAPDH, and the quantifications of pro-Caspase-3 to GAPDH and cleaved-Caspase-3 to GAPDH ratios. **(F)** The immunoblotting images of hepatic pro-GSDME, cleaved-GSDME and GAPDH, and the quantifications of pro-GSDME to GAPDH and cleaved-GSDME to GAPDH ratios. **(G)** The immunofluorescence of TUNEL staining (scale bars: 50 µm). n = 3 biologically independent samples in each group of immunoblotting images, ^*^p <0.05, ^**^p < 0.01, ^***^p < 0.001, ^****^p < 0.0001. The horizontal line indicates that the group on the left is compared with the group on the right.

Besides inducing pro-inflammatory cytokines expression, TLR4 signaling can also induce the transcription and translation of NLRP3 via NF-κB (29). Our data showed that H_2_ pretreatment reduced hepatic NLRP3 expression induced by LPS, and the low dose is better than the high dose (**Figure 7A**). In response to a wide variety of stimuli, NLRP3 can form a protein complex associated with the adaptor molecule ASC, and Caspase-1, named NLRP3 inflammasome (30, 31). The biochemical function of inflammasomes is to activate Caspase-1, which leads to the maturation of IL-1β and IL-18 and cleavage of GSDMD to induce pyroptosis, an inflammatory programmed cell death mode (32). Therefore, we further examined whether the effect of H_2_ on IL-1β and IL-18 cleavage is related to modulate NLRP3 inflammasome. Our immunoblotting showed that LPS enhanced the expression of ASC (**Figure 7B**), the levels of full length and cleaved Caspase-1 (**Figure 7C**), which were all reduced by H_2_ pretreatment (**Figures 7B, C**). The pharmacological rescue experiments by agonist (nigericin) and antagonist (MCC950) of NLRP3 further confirm the action of H_2_ on NLRP3 in vitro (**Figures 7D, E**), which indicated that NLRP3 is a key target of H_2_. The up-regulation of hepatic GSDMD protein levels and immunofluorescence intensity induced by LPS were all reduced by H_2_ pretreatment, and the low dose is better than that of high dose (**Figures 8A, B**). Besides, Caspase-11 (which is the homology of Caspase-4/5 in human) and Caspase-8 can also cleave GSDMD, and Caspase-3 can cleave GSDME, thus, lead to non-classical pyroptosis (33, 34). Our data showed that hepatic levels of both full length and cleaved forms of Caspase-8, -11 and -3, and GSDME, and fluorescence of TUNEL staining were all increased in LPS-challenged mice, both doses of H_2_ inhibited the expression and cleavage of Caspases and GSDME, and TUNEL staining, moreover, the high dose is more effective than the low dose (**Figures 8C–G**). Therefore, H_2_ pretreatment by intraperitoneal injection suppresses LPS-induced NLRP3 inflammasome activation and pyroptosis in the liver of LPS-challenged mice.

## 4 Discussion

H_2_ is a colorless, odorless, and the lightest and diffusible gas molecule. In 2007, Ikuroh Ohsawa et al. reported that exogenous H_2_ acts as a therapeutic antioxidant by selectively reducing cytotoxic oxygen radicals. Since then, many studies had shown supplement of exogenous H_2_ protected against acute or chronic damage of the liver, gallbladder, stomach, intestine, pancreas, brain, heart, lung, kidney, testis, ovary, breast, eye, ear, bones, and the skin, et al. (10). The methods of supplement of exogenous H_2_ primarily included inhalation of H_2_-O_2_ mixture, drinking H_2_-rich water, intraperitoneal injection of H_2_-rich saline (10). Here, we found that H_2_ pretreatment by intraperitoneal injection has a preventive effect on LPS-induced acute liver injury. It should be noted that our study utilized an LPS single-hit model, which is characterized by systemic endotoxemia and sterile inflammatory liver injury but does not fully recapitulate polymicrobial sepsis. Numerous animal models are available for the preclinical study of sepsis, and they fall into one of three general categories: (1) administration of exogenous toxins (e.g., LPS, zymosan); (2) virulent bacterial or viral challenge; (3) disruption of the host barrier, e.g., cecal ligation and puncture (CLP) or colon ascendens stent peritonitis (CASP) (35). Of the murine models used to study the pathophysiology of sepsis, CLP combines tissue necrosis and polymicrobial sepsis secondary to autologous fecal leakage, as well as hemodynamic and biochemical responses similar to those seen in septic humans (35). Therefore, the effect of intraperitoneal injection of H_2_ on bacterial sepsis requires further validation in more complex models, such as CLP animal models.

Similarly, we and others had previously reported that intraperitoneal injection of H_2_ can improve LPS-induced cardiac dysfunction (11) and acute alcohol-induced liver injury in mice (36), and reduce cerebral ischemia-reperfusion injury and improve the prognosis of cardiopulmonary cerebral resuscitation in a rabbit model of cardiac arrest (37). These studies demonstrated the protective effects of intraperitoneal injection of H_2_ on major organs. Inhalation H_2_ requires the specialized equipment and has the risk of combustion or explosion, and the H_2_-loading capacity of H_2_-rich water or H_2_-rich saline is relatively limited. In contrast, H_2_ intraperitoneal injection can mitigate these shortcomings; intraperitoneal injection of H_2_ can decrease the levels of TNFα, IL-1β, and IL-18 in serum (11), however, systematic studies on the systemic effects of H_2_ intraperitoneal injection, such as on organ indices, remain lacking. The endogenous H_2_ can also protect the liver against injuries, for example, enhanced H_2_ generation in intestine by feeding diet with 20% high amylose cornstarch alleviated hepatic I/R injury in rats (38); the gut microbiota derived H_2_ improved Concanavalin A (Con A)-induced hepatitis, while inhibition of gut microbiota by antibiotics enhanced Con A-induced hepatitis (39). Therefore, is the level of anti-infectious immunity of different individuals related to endogenous H_2_ levels? If so, can sepsis be improved by increasing endogenous H_2_ levels? 

LPS induced the production of intracellular ROS, which was required for TLR4-mediated innate immunity (40). When the septic acute liver injury occurs, excessive amounts of ROS are produced by the damaged mitochondria of hepatocytes (41) and by the resident immune cells (42). H_2_ is a therapeutic antioxidant by selectively reducing cytotoxic oxygen radicals (43). Therefore, we investigated the effects of H_2_ on hepatic oxidative stress in response to LPS. Oxidative post-translational modification of proteins by molecular oxygen (O_2_)- and nitric oxide (•NO)-derived reactive species is a usual process that occurs in mammalian tissues under both physiological and pathological conditions and can exert either regulatory or cytotoxic effects (44). Reactive-nitrogen species (RNS) such as peroxynitrite (ONOO^−^), that is, the reaction product of ^•^NO and superoxide (O_2_^−•^), nitryl chloride (NO_2_Cl) and ^•^NO_2_ react with the activated aromatic ring of tyrosine to form 3-NT. This modification occurs to both free form of amino acid (i.e., soluble/free tyrosine) and to tyrosine residues covalently bound within the backbone of peptides and proteins (45). Both immunoblotting and immunofluorescence staining showed that LPS increased hepatic 3-NT levels, in contrast, these were all suppressed by H_2_ pretreatment. This was similar to Ikuroh Ohsawa’s report, as that H_2_ selectively reduced the hydroxyl radical, the most cytotoxic of ROS (43). High level of ROS can inflict direct damage to lipids and form lipid peroxides, for example, MDA (23). The hepatic MDA are increased by LPS, which were reversed by H_2_. GSH quenches oxidizing substances (such as reactive hydroxyl free radicals, peroxynitrite, and H_2_O_2_) directly or reduces H_2_O_2_ (or lipid peroxide (lipid-OOH)) to water (or the corresponding lipid alcohol (lipid-OH)) under the catalysis of glutathione peroxidase (GPX) (46). Meanwhile, reduced GSH is oxidized into glutathione disulfide (GSSG) (46). Our data showed H_2_ enhanced hepatic GSH levels reduced by LPS, which indicated that the anti-oxidative capacity was increased by H_2_. MPO is a leukocyte-derived heme-containing peroxidase that can catalyze the formation of ROS, including hypochlorous acid (HOCl) (47, 48). Although MPO-derived ROS play a key role in neutrophil antimicrobial activity and host defense against various pathogens primarily by participating in phagocytosis, the elevated MPO levels are also associated with inflammation and increased oxidative stress (49). The increased hepatic MPO suggested excessive activation of immune cells in the liver, and H&E staining also supports this, while H_2_ pretreatment reduced hepatic MPO levels. Therefore, intraperitoneal injection of H_2_ can alleviate LPS-induced hepatic oxidative stress. 

The canonical signal transduction induced by LPS is the innate immune signaling mediated by TLR4, which is a classic receptor of LPS (27). All TLRs, except TLR3, use MyD88 as an adaptor protein (27). In MyD88-dependent TLR4 signaling, the E3 ubiquitin ligase TRAF6 is essential for TAB-TAK1 activation and subsequent NEMO-IKKα/β-NF-κB activation, while the released TRAF6 signaling complex in the cytoplasm leads to MAPK-AP-1 activation (27). Our results showed that the expression of hepatic TLR4 and phosphorylation of hepatic IKK, NF-κB p65, ERK1/2, p38 MAPK, and JNK were increased by LPS, and these were all reduced by H_2_. H_2_ seems to inhibit the activation of most signaling molecules detected in this study on TLR4-mediated signaling. However, innate immune signaling is a complex cascade, it is not clear that whether these signaling molecules are directly inhibited by H_2_, or indirectly by modulating one or more negative regulators of the innate immune signaling (50), or primarily through its established role as a selective antioxidant (43), thereby indirectly dampening redox-sensitive signaling nodes (e.g., in TLR4 signaling or inflammasome). These are worthy of investigation in the future.

The activation of transcription factor, such as NF-κB, results in up-regulation of pro-inflammatory cytokines, such as TNF-α, IL-1β, and IL-18 (27). As the inhibition of upstream of TLR4 by H_2_, there is no doubt that H_2_ can reduced the levels of TNF-α, pro-IL-1β, and pro-IL-18. We have previously reported that H_2_ can decrease the mRNA levels of pro-inflammatory cytokines TNFα, IL-1β, and IL-18 in LPS-challenged mice (11). More interesting, we found that the maturation of both IL-1β and IL-18 in the liver were also suppressed by H_2_. The cleaved Caspase-1 is responsible for cleaving pro-IL-1β and pro-IL-18 to form the maturation ones (30). The activated Caspase-1 is a canonical element in inflammasome, such as NLRP3 inflammasome consisting of NLRP3, ASC and Caspase-1 (30, 31). In this study, LPS increased the expression of NLRP3, ASC, and both full length and cleaved Caspase-1, while H_2_ displayed the inhibitory effect on these above, which indicated that H_2_ can inhibit the activation of NLRP3 inflammasome. The activated Caspase-1 can also cleave and activate GSDMD to induce an inflammatory programmed cell death, that is pyroptosis (51). Our data showed that H_2_ can decrease both the full length and cleaved GSDMD, and immunofluorescence staining further confirmed the inhibitory effect of H_2_ on GSDMD. This was like the data from Yang’s group, which indicated that H_2_ inhalation ameliorates cardiac remodeling and fibrosis by suppressing NLRP3 inflammasome-mediated pyroptosis (52–54). Human Caspase-4 and the mouse homologue Caspase-11 and human Caspase-5, can directly bind to LPS and lipid A with high specificity and affinity (33). In addition to Caspase-1, cleaved Caspase-8 and Caspase-11 also activate GSDMD to induce a non-canonical pyroptosis signaling (33), and Caspase-3 has been shown to cleave GSDME to induce another non-canonical pyroptosis (34). LPS enhanced these non-canonical pyroptosis signals in the liver, while the expression and activation of these proteins were reversed by H_2_. Moreover, the inhibited effects of H_2_ on cell death in the liver were further confirmed by TUNEL staining. These highlight that H_2_ intraperitoneal injection inhibited both the canonical pyroptosis signaling in the liver mediated by NLRP3 inflammasome to GSDMD, and the non-canonical pyroptosis signaling in the liver elicited by Caspase-8/11 to GSDMD and Caspase-3 to GSDME in LPS-challenged mice.

In conclusion, H_2_ pretreatment *via* intraperitoneal injection alleviated LPS-induced acute liver injury in mice through inhibiting hepatic oxidative stress, TLR4-IKK-NF-κB and -MAPKs innate immune signaling, NLRP3 inflammasome activation, and GSDMD/GSDME-elicited pyroptosis signaling. There are interactions between these signals, H_2_ can inhibit these signals, especially NLRP3, and this inhibition plays a key role in improving hepatocyte viability. However, is the effect of H_2_ on these signals independent, or is it achieved by modulating a key signaling molecule? In the future, research on key targets of H_2_ still needs to be strengthened.

## Data Availability

The raw data supporting the conclusions of this article will be made available by the authors, without undue reservation.
